# Non-Invasive Reproductive Hormone Monitoring in the Endangered Pygmy Hog (*Porcula salvania*)

**DOI:** 10.3390/ani11051324

**Published:** 2021-05-06

**Authors:** Vinod Kumar, Shyamalima Buragohain, Parag Jyoti Deka, Goutam Narayan, Govindhaswamy Umapathy

**Affiliations:** 1 Laboratory for the Conservation of Endangered Species, CSIR-Centre for Cellular and Molecular Biology, Uppal Road, Hyderabad 500007, India; vinod@ccmb.res.in (V.K.); shy20aug@gmail.com (S.B.); 2 Durrell Wildlife Conservation Trust, Pygmy Hog Conservation Programme (PHCP), Indira Nagar, Basistha, Guwahati, Assam 781029, India; parag.deka@durrell.org; 3 Aaranyak, Threatened Species Recovery Programme (TSRP), 13, Tayab Ali Byelane, Bishnu Rabha Path, Beltola, Guwahati 781028, India; 4 EcoSystems-India, Rare & Endangered Species Conservation Unit (RESCU), A-2 Florican Enclave, H.No.3, Basisthapur Bylane No.2, Beltola, Guwahati, Assam 781028, India; Goutam.Narayan@gmail.com

**Keywords:** pygmy hog, *Porcula salvania*, progesterone, testosterone, fecal hormone, pregnancy detection, Assam

## Abstract

**Simple Summary:**

The pygmy hog is one of the world’s rarest suids and classified as an endangered species. Efforts are being made to breed them in captivity and reintroduce them into the wild. In this study, we examined reproductive hormones in captive pygmy hogs using a non-invasive method by collecting 785 fecal samples from five females and two males for 12 months. High-pressure liquid chromatography was performed to examine the presence of immunoreactive progesterone and testosterone metabolites in the fecal samples. We standardized and validated enzyme immunoassays (EIA) for fecal progesterone and testosterone metabolites. Using progesterone EIA, we were able to detect pregnancies in four females and estimate the relevant gestation period. We also recorded 172 births from the captive breeding center and found strong seasonality patterns in births. In males, fecal testosterone metabolite concentrations were higher in the breeding season than in the non-breeding season as evidenced by elevated testosterone concentrations during breeding season. A significant difference in fecal progesterone metabolites concentration was observed between non-pregnant and pregnant females. This study can directly help in monitoring the reproductive status of reintroduced hogs both in the wild and in conservation breeding programs in India and elsewhere.

**Abstract:**

The pygmy hog (*Porcula salvania*), until recently was classified as a critically endangered suid facing the threat of extinction due to habitat degradation. Efforts are being made to protect the pygmy hog from extinction and breed them in captivity under the Pygmy Hog Conservation Programme (PHCP). However, very little information is available on the reproductive physiology of pygmy hogs. Therefore, the present study aims to standardize enzyme immunoassays (EIAs) for monitoring pregnancy and reproductive status using progesterone and testosterone metabolites. A total of 785 fecal samples were collected from five females and two males over a period of one year from the PHCP Research and Breeding Centre, Guwahati, Assam. High-pressure liquid chromatography (HPLC) analysis revealed the presence of immunoreactive progesterone and testosterone metabolites in feces. Mating was observed in all five females, and four of them gave birth successfully. We were able to detect pregnancy using fecal progesterone metabolites. The mean gestation period, based on mating and parturition, was estimated to be 153.25 days from the four females studied. The breeding center recorded 172 births between 1996 and 2000 and found strong seasonal patterns in the birth rate, with most of the births occurring between May and June. In the males, fecal testosterone metabolites were significantly higher in the breeding season than in the non-breeding season. This is the first study on the subject and will help with future breeding programs in other captive breeding centers and with reproductive monitoring of reintroduced populations.

## 1. Introduction

The pygmy hog (*Porcula salvania*) is the world’s rarest and smallest wild suid belonging to the family Suidae [1]. It was listed as critically endangered by the IUCN Red List until 2019, but it has recently been downgraded to endangered [2] due to the conservation breeding and reintroduction efforts of the Pygmy Hog Conservation Programme (PHCP). It continues to be listed under Schedule I of the Indian Wildlife (Protection) Act, 1972. The pygmy hog is considered an indicator species of the healthy grassland ecosystem andalso suffers from poor wildlife management practices, persistent burning and other anthropogenic disturbances [3,4]. Once widespread across tall wet grassland in a narrow strip south of the Himalayan foothills from Uttar Pradesh to Assam (India) across Nepal and Bhutan, the pygmy hog population declined in the last century. By the early 1990s, it was reduced to a single global population of 400–500 individuals in the Manas National Park, India. The pygmy hog population has declined due to degradation and loss of grassland, the rapid expansion of human settlements and agricultural encroachments, flood control schemes, and improper management of grassland ecosystems [5,6,7]. Furthermore, planting trees in grasslands andthe indiscriminate use of fire to create an opening and to promote fresh grass are other major threats to the pygmy hog’s habitat [8]. Interestingly, pygmy hog habitats are shared by other endangered animals that include the one-horned Indian rhinoceros (*Rhinoceros unicornis*), tiger (*Panthera tigris*), hispid hare (*Caprolagus hispid*), water buffalo (*Bubalus arnee*), Bengal florican (*Houbaropsisbengalensis*), and Assam roofed turtle (*Kachugasylhetensis*).

Efforts are being made to save the species from extinction, which include conservation breeding and reintroduction. Initial efforts in 1971 and 1976 failed to yield any success due to the nonscientific method of breeding [5]. In 1996, the Assam Forest Department, Durrell Wildlife Conservation Trust, and IUCN/SSC Wild Pig Specialist Group, along with Eco-Systems-India, set up a research and breeding center to breed pygmy hogs in captivity and release them into the wild to replenish natural populations. Until 2020, the program had successfully produced 683 individuals from tenwild-caught originalpygmy hogs. Between 2008 and 2018, a total of 116 captive-bred individuals were released periodically into three reintroduction sites at SonaiRupai Wildlife Sanctuary, Rajiv Gandhi Orang National Park, and Barnadi Wildlife Sanctuary in Assam [8]. In 2020, 14 hogs were released in the eastern ranges of Manas National Park, where less than 100 hogs may now survive in the central range. Thus, 130 captive-born individuals have been released into the wild as part of the continuing recovery program, which has put increased stress on the efforts to restore and manage suitable grasslands in their former range.

Pygmy hogs eat a wide range of food, including roots, tubers, shoots, insects, earthworms, eggs, and carrion. They are foragers and spend six to eight hours searching for food by digging and turning up litter and topsoil using their snout [4]. They live in groups of 4–6 individuals, primarily adults with their young. Adult males weigh about 8–10 kg with a head-body length of 61–71 cm, while females weigh 6–8 kg with a head-body length of 55–62 cm [6]. Most of the mating in captivity occurred between December and February, and births were recorded before the monsoon (May to September). The litter size ranged between 2–7 but was mostly in the range of 4–6 in captivity [6].

Reproductive seasonality is characteristic of many mammalian species. However, seasonality is a result of various intricate factors formed by physiological mechanisms. The physiology of species is significantly influenced by environmental factors such as climate, temperature, humidity, photoperiod, nutrition, foraging conditions, and social interactions between the conspecifics [9,10,11]. The physiological control of seasonal breeding is driven by the central circadian regulatory system situated in the suprachiasmatic nucleus (SCN), which involves modulation of the neuroendocrine mechanism using the hypothalamus and the pituitary and pineal glands to regulate the breeding season. Most species show strong seasonal reproductive variation evidenced by increasing levels of sex steroids, including long-tailed macaques (*Macacafascicularis*) [12], plains zebras (*Equus quagga*),springboks (*Antidorcas marsupialis*) [13], Iberian red deer (*Cervus elaphushispanicus*) [14], Père David’s deer (*Elaphurusdavidianus*) [15], coyotes (*Canis latrans*) [16], and camels (*Camelus dromedarius*) [17]. The wild boar (*Sus scrofa*), a close relative of the pygmy hog, exhibits seasonal polyestrous, while the domestic pig is known to breed throughout the year [18].

Understanding basic reproductive function is crucial for successful conservation breeding programs of endangered species, and it can be studied by monitoring circulating hormones [19,20,21]. Hormones can be measured in a variety of biological samples such as feces [22,23,24], urine [25], blood [26], saliva [27], milk [28], and hair [29,30,31]. Although circulating hormones in the blood give an accurate measurement, blood sampling for long-term monitoring of wild animals is challenging and stressful. As an alternative method, estimating the hormone metabolites in feces as a non-invasive method is feasible since circulating hormones metabolize in the liver and are excreted through the feces. In many species, no, or very little, native hormone is present in the feces, and most of the hormones are metabolized. The excreting metabolites vary considerably between even closely related species and in some species even between the sexes. Therefore, each assay needs to be validated with biologically relevant concentrations of the hormone and its metabolites in the feces [20]. Fecal steroid analysis has been used to assess the reproductive status and endocrine function in various captive and free-ranging wild species, including Asian elephants [32,33], musk deer [34], red pandas [35], primates [36,37], big cats [22,38], birds [39], and chelonians [40].

Despite the successful breeding program, the reproductive physiology of this species is poorly understood; the ongoing conservation breeding program provides an exceptional opportunity to understand the reproductive biology of the species, particularly reproductive physiology of endangered suids. The present study aimed (1) to characterize fecal hormone metabolites using high-pressure liquid chromatography (HPLC), (2) to biologically validate enzyme immunoassays for progesterone and testosterone metabolites, (3) to monitor the pregnancy and reproductive status in captive pygmy hogs using fecal steroid hormone analysis, and (4) to examine the seasonality of reproduction. This is the first report on monitoring the reproductive status of pygmy hogs in India using a non-invasive method.

## 2. Materials and Methods

### 2.1. Sample Collection

A total of 785 fecal samples were collected from seven captive pygmy hogs (two males and five females) from the Pygmy Hog Research and Breeding Center, Basistha, Guwahati, Assam. Males and females were caged separately and adjacent to each other. The males were allowed into the female enclosures during the breeding season (based on 10 y of breeding and mating records in the center) for mating. The captive pygmy hogs were fed daily with a balanced diet with a wide range of variety of tubers, cereals, pulses, fruits, vegetables, and eggs. Furthermore, they were allowed to forage for natural vegetation and soil invertebrates such as earthworms, termites, ants, and beetles. The enclosures were planted with *Saccharumnarenga* and *Phragmitieskarka* grasses, which are known to occur in pygmy hog habitats. The temperatures in this region range from 11° C (January) to 33 °C (July), and June to September is the rainy season with peak rainfall during July.

Samples were collected three to four days in a week during one year (July 2015 to July 2016). Due to space restrictions, samples collected for some individuals were discontinued over some periods. Freshly collected fecal samples were dried in a hot air oven at 70 °C; pulverized; and stored in zip lock bags with date, individual IDs, etc. at 4 °C until further extraction. Observations, if any, on mating and other reproductive behaviors (nudging, mounting, squeaking, soft grunting) were recorded on a daily basis during the sample collection period. Details of the age, sex, individual IDs and the number of samples collected are given in Table 1.

### 2.2. Birth and Gestation Data

To examine the seasonality of births in pygmy hogs, the data on births from April 1996 to July 2020 at the Pygmy Hog Research and Breeding Centre, Basistha, Guwahati, Assam were collected and analyzed. Data on mating observations and parturition were also collected from the center’s records for estimating the length of gestation.

### 2.3. Extraction of Fecal Steroid Metabolites

Fecal samples were extracted using the previously described procedure with minor modifications [41,42]. The dried fecal powder was sieved and weighed to 0.2 g in a 15 mL falcon tube, and 2 mL of 80% methanol was added and vortexed for 20 min. Furthermore, samples were then kept at 4 °C overnight and centrifuged at 2000× *g* for 10 min, and supernatants were stored in −20 °C for further analysis.

### 2.4. Hormone Assays

Fecal progesterone was measured using the monoclonal anti-progesterone antibody (CL425; provided by Dr. Coralie Munro, University of California, Davis, CA, USA). The progesterone antibody had 100% cross-reactivity with progesterone and a variety of 5α- and β-reduced pregnane [43]. Fecal testosterone was measured using the polyclonal anti-testosterone antibody (R156/7; provided by Dr. Coralie Munro, University of California, Davis, CA, USA). The testosterone antibody had 100% cross-reactivity with testosterone; 57.4% with dihydrotestosterone; <0.3% with androstenedione; and <0.1% with androsterone, dihydroepiandrosterone, β-estradiol, and progesterone [44].

### 2.5. Enzyme Immunoassay Procedure

Enzyme immunoassays (EIAs) for fecal progesterone and testosterone were performed as described previously [32,34]. The 96-well Nunc–Maxisorp microtiter plate was coated with 50 µL of antibody per well, diluted in coating buffer (0.05 M sodium bicarbonate buffer, pH 9.6) and kept at 4 °C for overnight incubation. The plate was washed four times with washing buffer (0.15 M NaCl, 0.05% Tween 20). Added to each well was 50 µL of fecal extract diluted in EIA buffer (0.1 M PBS, pH 7, and 1% BSA) or standard followed by 50 µL of conjugated HRP (horseradish peroxidase), incubated at room temperature for 2 h. The plate was then washed 4 times with washing buffer; then, 50 µL of TMB (Tetramethyl benzidine/H_2_O_2_, Genei, Bangalore) was added to each well and kept in the dark for 5–10 min for color development. The reaction was stopped using 50 µL of stop solution (1M Hydrochloric acid (HCL), and optical density (absorbance) was then measured at 450 nm using an ELISA reader (Thermo Multiskan Spectrum Plate Reader, version 2.4.2; Thermo Scientific, Helsinki, Finland).

### 2.6. High-Performance Liquid Chromatography

To evaluate the immunoreactivity of fecal progesterone and testosterone with corresponding antibody and separation of fecal steroid metabolites, high-performance liquid chromatography was performed using the Shimadzu CTO-10AS system (Shimadzu Corporation, Tokyo, Japan). Steroid specific reverse-phase C-18 column was used (waters column, symmetry C-18, 4.6 3 20 mm, 3.5 mm, intelligent speed (IS)column to identify the steroid metabolites from fecal samples. Before HPLC analysis, pooled fecal extracts were passed through Sep-Pak C18 cartridges (Waters, Milford, MA, USA) for purification and eluted with 3 mL of absolute methanol. The purified fecal extracts were dried using nitrogen gas and resuspended in 100 µL of absolute methanol as described previously [34,45]. The protocol running time was 8 min using a gradient flow of 20–64% acetonitrile (ACN):water (H_2_O) at a flow rate of 1 mL/min, and steroid hormones were detected at the 190 to 400 nm wavelength. Fractions were collected from progesterone and testosterone standards and pooled fecal extracts manually, about 250 µL every 15 s (4 fractions/minute), and vacuum dried. The dried fractions were resuspended in 100 µL of EIA buffer and used in the assay.

### 2.7. Data Analysis

Data are represented as mean ± standard error of the mean. Correlation analysis for parallelism was carried out using Pearson’s correlation analysis. The fecal testosterone metabolite data are presented using descriptive statistics because of the small sample size. The differences between mean progesterone metabolite concentrations of pregnant and non-pregnant samples were analyzed using a Wilcoxon signed-rank test, as data were not normally distributed (using Shapiro–Wilk test). All statistical analyses were carried out using SPSS 17.0.

## 3. Results

### 3.1. Enzyme Immunoassay Validation

**Figure 1:**
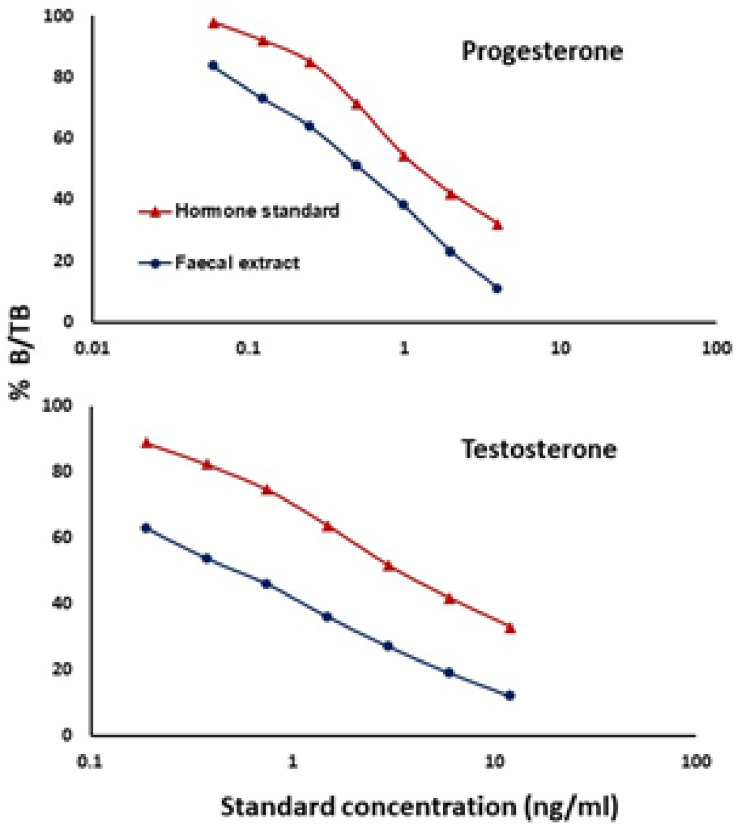
Parallelism between the serial dilution of pooled fecal extracts of pygmy hogs (circles) and the respective standards (triangles) of progesterone and testosterone. The Y axis show percentage binding/total binding (% B/TB) and X axis show standard concentration (ng/mL).

**Figure 2:**
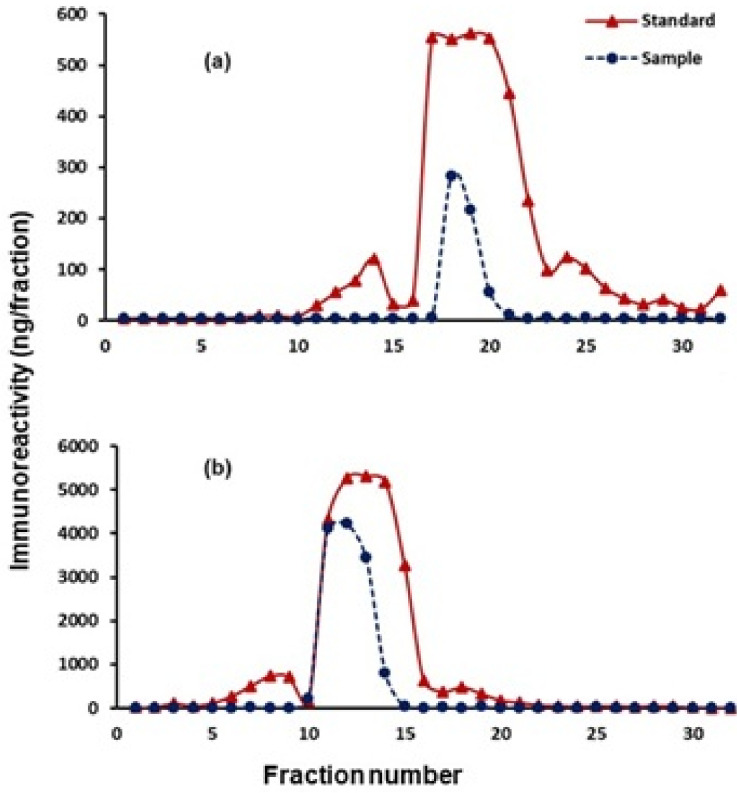
HPLC profiles of immunoreactive fecal progesterone (**a**) and testosterone (**b**) in pygmy hogs.

Progesterone and testosterone enzyme immunoassays were validated by demonstrating the parallel displacement curves between the pooled serial dilution ofpygmy hog fecal extracts and their respective standards to determine the immunological activity of fecal hormone and standard with the corresponding antibodies used in the assays (Figure 1). Assay sensitivity was calculated with 90% binding and found to be 0.39 and 1.17 pg./well for progesterone and testosterone, respectively. The intra- and inter-assay coefficient of variations (CV) were 6.6 and 11.6% for progesterone and 6.8 and 11.1% for testosterone. Recovery and accuracy of a known amount of unlabeled steroid hormones in fecal extracts were 81.4 ± 4.4% for progesterone and 83.1 ± 11.42% for testosterone. The correlation (r^2^) and slope (m) values for the recovered exogenous steroids were r^2^ = 0.99, m = 0.87 and r^2^ = 0.98, m = 0.80 for progesterone and testosterone, respectively. The presence of fecal progesterone and testosterone confirmed by HPLC profiles and eluted fractions showed the immunoreactivity with corresponding EIAs. However, fecal extracts showed a single large peak due to close immunoreactivities. Moreover, standard concentrations of progesterone and testosterone were higher as compared to pooled fecal steroid metabolites (Figure 2).

### 3.2. Reproductive Monitoring

A total of 785 samples were collected from five adult females and two adult males for a one-year period. All five females were found mating with males between January and April, and four of them delivered young, although one died due to unknown reasons (PH368) (Table 1).

**Figure 3:**
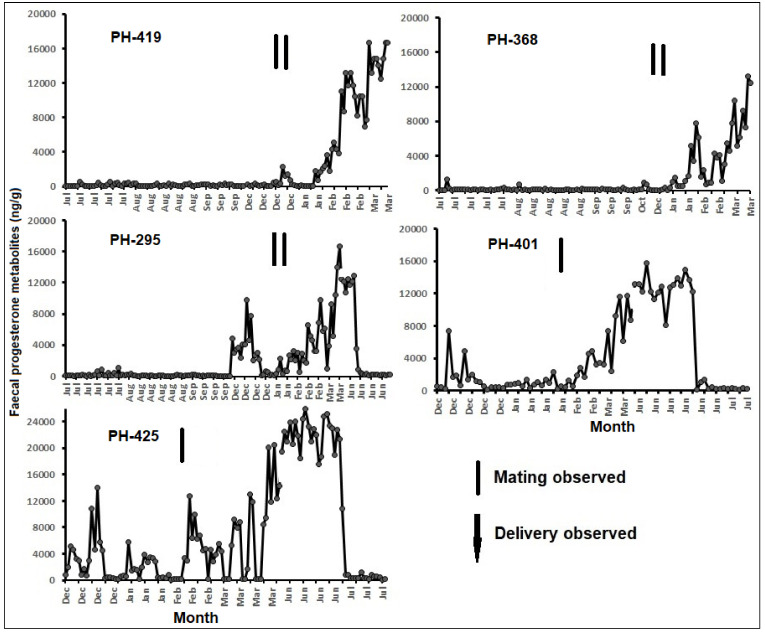
Fecal progesterone metabolite concentrations in five females monitored over 10 to 12 months at the Pygmy Hog Conservation Programme (PHCP), Guwahati (vertical bars—mating observed; down arrow—delivery of piglets observed). PH (Pygmy Hog)-419 could not be sampled before and after the delivery due to restriction in space, while PH (Pygmy Hog)-368 had died due to unknown reasons.

**Figure 4:**
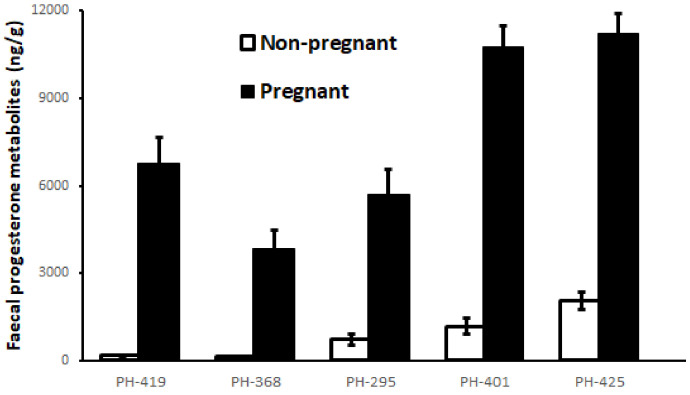
Fecal progesterone metabolite concentrations in pregnant and non-pregnant Pygmy Hog (PH) individuals (*n* = 5 females; 523 samples). The pregnant samples included two days after successful mating until delivery, while the non-pregnant samples included non-pregnant periods.

Overall, individual fecal progesterone metabolite concentrations ranged from 172 to 2590 ng/g (Figure 3). The pregnant females had significantly higher fecal progesterone metabolite concentrations compared to their non-pregnant values (Wilcoxon signed rank test, *p* < 0.001 for all five animals; Figure 4). All the pregnant females showed similar progesterone profiles throughout their pregnancies.

Based on observation of mating and parturitions of four females, the gestation period ranged from 148 to 157 days with an average of 153.25 days, which correlates to previous data recorded by the breeding center that ranged from 148 to 161 days with a mean of 154.40 days (*n* = 30).

**Figure 5:**
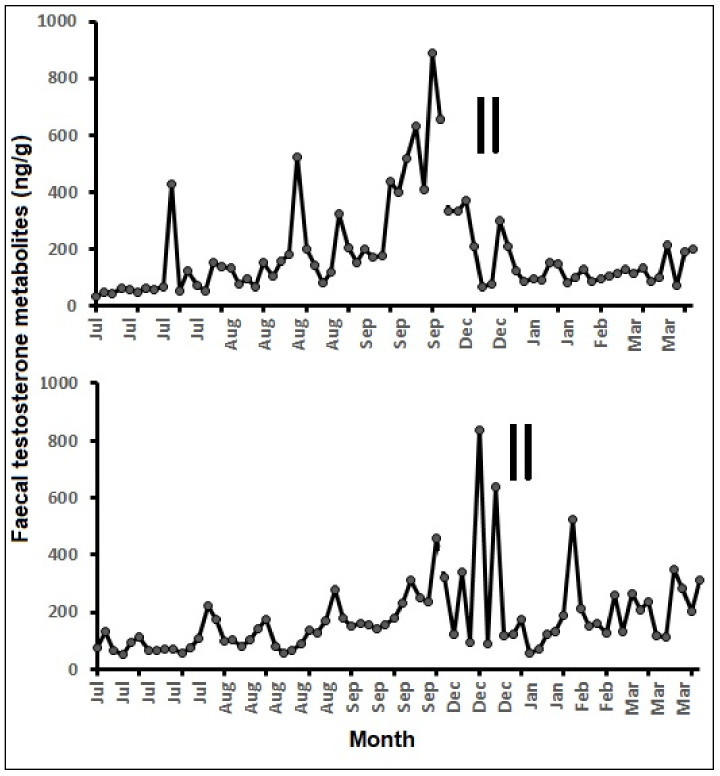
Fecal testosterone metabolite concentrations of two males monitored at PHCP, Guwahati (vertical bars—mating observed).

Two adult males, those involved in successful mating with the females, were also monitored for fecal testosterone metabolites, and they showed elevated fecal testosterone concentrations between September and December (Figure 5), which is about two to three months before the mating observations. Overall, the fecal testosterone metabolite concentrations ranged from 36 to 888 ng/g, and the elevated values were recorded during the pre-mating period (September–December).

**Figure 6:**
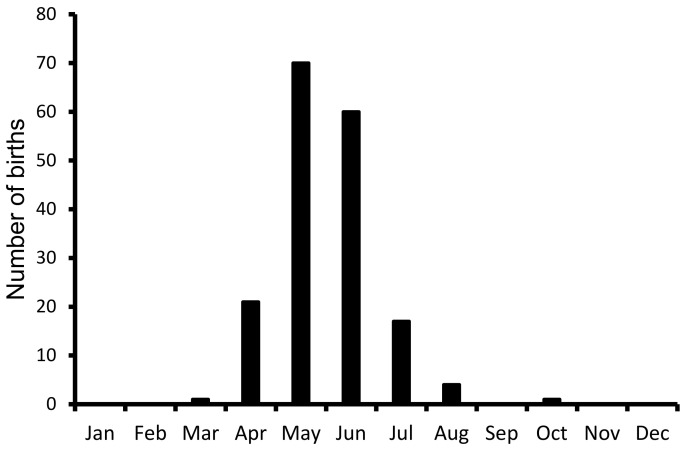
Distribution of births of pigmy hogs in PHCP, Guwahati. About 172 births were recorded between April 1996 and July 2020, including those of three wild-caught females.

A total of 172 births were recorded between 1996 and 2020 in the PHCP, Guwahati. All of the births were recorded between March and October, and about 74.71% of births were observed in May and June, showing a strong seasonality in the births (Figure 6).

## 4. Discussion

The present study reports on the standardization of enzyme immunoassays (EIAs) for fecal progesterone and testosterone metabolites and the endocrine patterns of reproductive hormones in endangered captive pygmy hogs using a non-invasive method. For the firsttime, long-term monitoring of the reproductive hormones in pygmy hogs was undertaken in a captive population. As expected, we found immunoreactive progesterone and testosterone metabolites in fecal samples of pygmy hogs using HPLC analysis. The progesterone metabolites in the fecal extracts could be monitored using a monoclonal antibody EIA (CL425) developed against progesterone (UC Davis, USA). This antibody reported high cross-reactivity with five alpha and beta pregnane metabolites excreted in the feces of a variety of species [43]. The progesterone EIA (CL425) has been previously standardized to detect pregnancy in a wide range of animals, such as the Himalayan musk deer (*Moschus chrysogaster*) [34], dugong (*Dugong dugon*) [46], maned wolf (*Chrysocyonbrachyurus*) [47], black rhinoceros (Dicerosbicornis) and white rhinoceros (*Ceratotheriumsimum*) [48], giant anteater (*Myrmecophagatridactyla*) [49], giraffe (*Giraffa camelopardalisrothschildi*) [50], Nile hippopotamus (*Hippopotamus amphibius*) [51] and red brocket deer (*Mazama americana*) [52]. In this study, we were able to successfully monitor pregnancies in pygmy hogs and were also able to distinguish between pregnant (>3000 ng/g) and non-pregnant values using fecal progesterone. This finding has a direct implication on the successful breeding and monitoring of reproduction in one of the most endangered mammals in the world.

Of the five females, four were observed to successfully mate and conceive, as evidenced by the delivery of their litters (size = 3–4). One of the pygmy hogs (PH 368) died during the study period and was found to be pregnant, as four fetuses were discoveredduring the post-mortem. The mean gestation period was estimated to be 153.25 days based on mating and delivery observations. During pregnancy, the fecal progesterone metabolite concentrations were elevated in all females until parturition. The fecal progesterone concentrations dropped to baseline values within a few days of parturition. The observed gestation period was also within the range of data from the breeding center, which was between 148 to 161 days from 30 females studied. However, previous reports suggested that the mean gestation period was 120 ± 5 days, and it ranged between 110 and 130 days based on the behavioral observations [53,54,55]. Interestingly, the gestation periods in the Suidae family ranges widely from 115 days in wild boar (*Sus scrofa*) to 170 days in the common warthog (*Phacochoerus africanus*) [56]. The present observation is within the range of the Suidae family’s gestation period. Furthermore, the present study shows that pygmy hogs are seasonal breeders as evidenced by most of the births being recorded within a few months before the monsoon, while their related species, the domestic pig, in this region breeds throughout the year [18].

Previously, testosterone EIA (polyclonal antibody, R156/7) has been reported for monitoring fecal testosterone metabolites in a wide range of animals, including the pronghorn (*Antilocapra americana peninsularis*) [57], red river hog (*Potamochoerusporcus*) [58], and polar bear (*Ursus maritimus*) [59]. Fecal testosterone metabolite levels of two monitored pygmy hogs did not show aclear cycle; however, there were elevated concentrations during the September–December period for both males. Most of the mating was observed between December and February, which is about one to two months after the elevated fecal testosterone metabolites in males. Fecal testosterone metabolites elevation in mammals is directly related to reproductive preparedness and sperm production. Overall, the elevated testosterone metabolite concentrations were related to male fitness in breeding, as evidenced by mating with the females during December and January.

Previous studies have shown that the analysis of fecal steroid metabolites could be conductedin other members of the Suidae family, including the red river hog (*Potamocherusporcus*), common warthog (*Phacochoerus africanus*), babirusa (*Babyrousababyrussa*) [60] wild boar (*Sus scrofa*) [18], and collared peccary (*Pecaritajacu*) [61]. However, this is the first report of the validation and standardization of enzyme immunoassays (EIAs) for reproductive monitoring in pygmy hogs using non-invasive methods. Since the pygmy hog is considered one of the most endangered mammals globally, this study maydirectly help breeding management in captivity. Furthermore, this method could be used forfertility monitoring and pregnancy detection in pygmy hogs in captivity and in the wild as well as inreintroduced populations.

## 5. Conclusions

This is the first study on reproductive hormone (progesterone and testosterone) monitoring in endangered pygmy hogs using a non-invasive method. Fecal progesterone and testosterone EIAs can be used to detect the pregnancy and fertility status in pygmy hogs. This study mayfurther facilitate reproductive monitoring of breeding programs in captivity and also assist in the management of wild and reintroduced populations.

## Figures and Tables

**Table 1:** Details of the animals studied, samples collected, mating, parturition, and gestation period of pygmy hogs at the Pygmy Hog Research and Breeding Center, Guwahati, Assam.

S. No	ID of the Animal	Sex	Age of the Animal (Years on January 2016)	No. of Samples Collected	Mating Dates	Date of Parturition	Gestation Period (Days)
1	PH 419	Female	4	141	23 December 2015	20 May 2016	148
2	PH 368	Female	4.3	124	26 December 2015	Died	
3	PH 295	Female	6	159	02 January 2016	16 June 2016	157
4	PH 401	Female	3	84	15 January 2016	18 June 2016	153
5	PH 425	Female	2	124	31 January 2016	03 July 2016	155
6	PH 418	Male	4	77			
7	PH 294	Male	6	76			
